# Fluid and White Matter Suppression contrasts MRI improves Deep Learning detection of Multiple Sclerosis Cortical Lesions

**DOI:** 10.1016/j.nicl.2025.103818

**Published:** 2025-07-14

**Authors:** Pedro M. Gordaliza, Jannis Müller, Alessandro Cagol, Nataliia Molchanova, Francesco La Rosa, Charidimos Tsagkas, Cristina Granziera, Meritxell Bach Cuadra

**Affiliations:** a CIBM Center for Biomedical Imaging, Lausanne, Switzerland; b Radiology Department, Lausanne University and University Hospital, Lausanne, Switzerland; c Translational Imaging in Neurology (ThINk), Department of Biomedical Engineering, Faculty of Medicine, Basel, Switzerland; d University Hospital Basel and University of Basel, Neurologic Clinic and Policlinic, MS Center University Hospital, Basel, Switzerland; e Research Center for Clinical Neuroimmunology and Neuroscience Basel (RC2NB), Basel, Switzerland; f Dipartimento di Scienze della Salute, Università degli Studi di Genova, Genova, Italy; g Institute of Informatics, University of Applied Sciences and Arts of Western Switzerland (HES-SO), Sierre, Switzerland; h Windreich Department of Artificial Intelligence and Human Health, Department of Neurology, Icahn School of Medicine at Mount Sinai, New York, United States; i Translational Neuroradiology Section, Division of Neuroimmunology and Neurovirology, National Institute of Neurological Disorders and Stroke, National Institutes of Health (NIH), Bethesda, United States

**Keywords:** Cortical Lesions, Assessment, Multiple Sclerosis, Deep Learning, MRI, FLAWS

## Abstract

**Purpose:**

To investigate the efficacy of Fluid and White Matter Suppression (FLAWS) MRI sequence in improving Deep Learning (DL)-based detection and segmentation of cortical lesions in Multiple Sclerosis (MS) patients even, and to develop models that can generalize to clinical settings where only standard T1-weighted images (MPRAGE) are available.

**Materials and Methods:**

In this multi-site study, we analyzed 204 MS patients using DL models developed with FLAWS and Magnetization Prepared 2 Rapid Acquisition Gradient Echoes (MP2RAGE) sequences. Reference standard annotations were established through two approaches: (1) consensus of three expert raters across all contrasts, and (2) single-rater annotations for individual modalities. Models were validated on both internal and external datasets, with performance assessed using $F_{1}$-score for detection and DSC for segmentation accuracy.

**Results:**

Models involving FLAWS demonstrated superior performance over MP2RAGE-only models. The model combining MP2RAGE and FLAWS achieved CL detection with median $F_{1}$-score of 0.667[0.339−0.840] compared to multi-rater consensus. Models trained on comprehensive consensus annotations outperformed those trained on single-modality annotations. Notably, a model trained on MP2RAGE but leveraging FLAWS-derived annotations showed strong generalization when applied to standard clinical Magnetization Prepared Rapid Gradient-Echo (MPRAGE) datasets from a different institution (median $F_{1}$-score: 0.55[0.211−0.998]), demonstrating successful knowledge transfer from advanced research sequences to routine clinical sequences.

**Conclusion:**

Integration of FLAWS-derived contrasts and annotations significantly improves DL-based CL detection and segmentation. The models demonstrate capability in identifying lesions missed by individual raters and maintain robust performance when applied to standard clinical sequences at external sites. This cross-sequence generalization facilitates immediate clinical translation, supported by publicly available inference models on DockerHub.

## Highlights

• MP2RAGE+FLAWS MRI sequences achieves superior cortical lesion detection performance.
• Quantitative validation shows median $F_{1}$ of 0.667[0.339−0.840] with combined sequences.
• FLAWS-trained model generalizes well to standard MPRAGE images ($F_{1}$: 0.55[0.211−0.998).
• Model demonstrates immediate clinical applicability on standard MPRAGE without FLAWS input.
• Implementation publicly accessible via DockerHub for widespread clinical adoption.

## Graphical abstract

**Figure:**
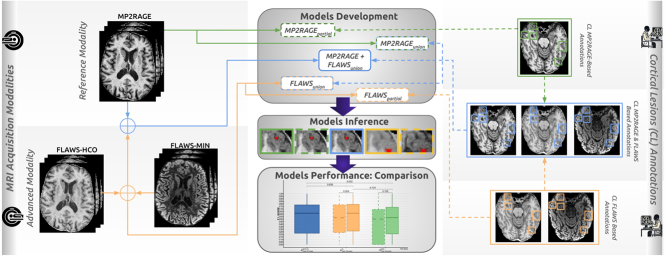

## 1 Introduction

Magnetic Resonance Imaging (MRI) is pivotal for profiling neurological disorders and indispensable for assessing those with complex etiology, such as Multiple Sclerosis (MS) (Filippi et al., 2019, Granziera et al., 2021). Disease-related imaging biomarker assessment provides insights into the underlying biological mechanisms driving these conditions. In MS, the most commonly employed imaging biomarkers are White Matter Lesions (WML), visualized as hyperintensities on T2-weighted contrasts. Despite their lack of sensitivity, WML dissemination in space and time is required for MS diagnosis according to *McDonald criteria* (Filippi et al., 2019, Thompson et al., 2018). Recent expert consensus, however, has indicated Cortical Lesions (CL) can provide increased specificity, as evidenced using advanced MRI techniques (Filippi et al., 2013, Beck et al., 2024, Cagol et al., 2024), verified through histopathology studies (Madsen et al., 2021). However, the inclusion of CL in routine assessments remains limited, primarily due to the lack of sensitivity in detecting these using standard clinical field strength scanners (1.5T or 3T) and conventional sequences (such as MPRAGE or T1-weighted imaging).

Enhancing CL detection necessitates the utilization of dedicated advanced multi-contrast MRI sequences and/or high-field scanners, offering greater sensitivity in gray matter and a finer spatial resolution (Maranzano et al., 2019). However, data of both natures are limited, hindering the development and application of Deep Learning (DL) methods (Hinton, 2018) to assist in the more clinically relevant identification of CL. The cost and time constraints associated with ultra high-field scanners confine their use to few research settings, where experts manually identify CL for specific trials, albeit with moderate inter-rater agreement (i.e., Cohen κ≈0.50−0.69) (Madsen et al., 2021).

Leveraging new advanced MRI sequences using clinical scanners offers a more widely accessible solution, despite shared challenges such as limited sensitivity, time-consuming manual evaluation and data scarcity. Contrasts such as Magnetization Prepared 2 Rapid Acquisition Gradient Echoes (MP2RAGE) (κ=0.582 in Müller et al. (2022)), Phase Sensitive Inversion Recovery, Magnetization Prepared Rapid Gradient-Echo (MPRAGE) or Double Inversion Recovery (DIR) (κ=−0.36 in Nielsen et al. (2012)) have been assessed to this end.

**Fig. 1:**
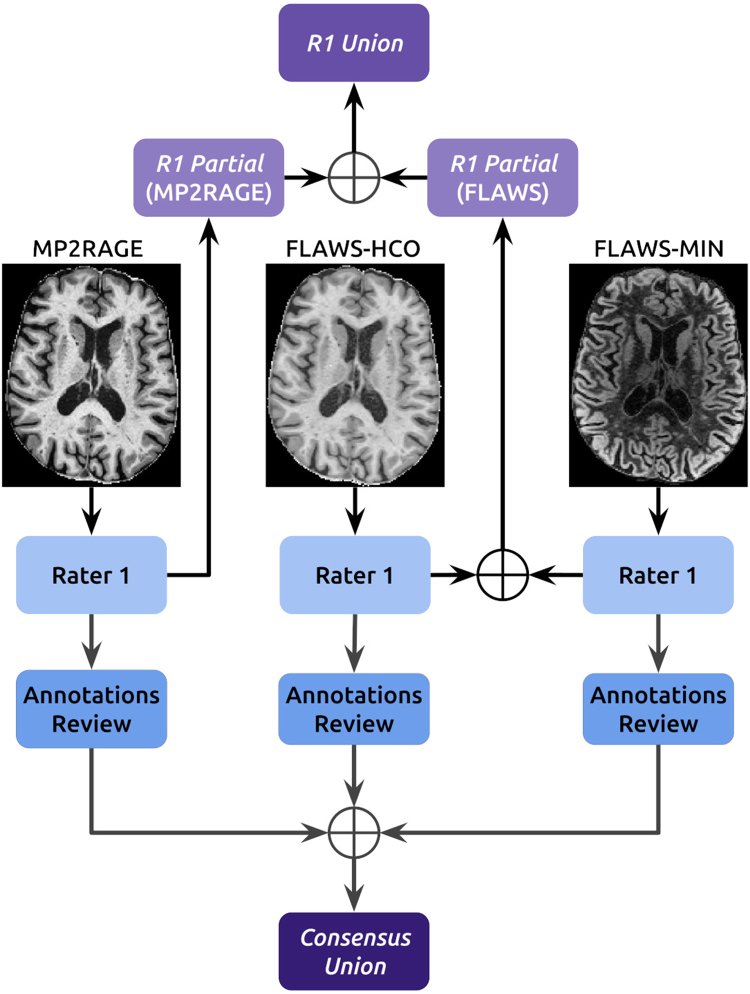
Schematic representation of the reference standard annotation process for *A-MP2+FLAWS* dataset. Three MRI sequences, Magnetization Prepared 2 Rapid Acquisition Gradient Echoes (MP2RAGE), Fluid and White Matter Suppression (FLAWS)-HCO, and FLAWS-MIN are independently annotated by Rater 1 (*R1*). These annotations are combined to form *R1 partial* annotations for MP2RAGE and FLAWS separately (providing the data partitions ${\text{A}}_{\text{Train or Test-R1partial}}^{\text{MP2+FL or MP2 or FL}}$), and *R1 Union* annotations combining all sequences (providing the data partitions ${\text{A}}_{\text{Train or Test-R1union}}^{\text{MP2+FL or MP2 or FL}}$). The initial annotations of 30 subjects undergo review, leading to the *Consensus Union* (providing the data partition ${\text{A}}_{\text{Test-Consensus}}^{\text{MP2+FL or MP2 or FL}}$) employed in Experiment 2.

A promising new sequence in this context is Fluid and White Matter Suppression (FLAWS) (Tanner et al., 2012). FLAWS provides both white matter and cerebrospinal fluid-suppressed 3D high spatial resolution contrasts simultaneously in one acquisition (*FLAWS1* and *FLAWS2*). These signals can be combined in a voxel-based manner to reconstruct multiple contrasts (i.e., ${\text{FLAWS}}_{HCO}$ for the high contrast images after WM signal suppression and ${\text{FLAWS}}_{MIN}$ for the minimum of both FLAWS. See Fig. 1, Fig. 5), showing higher sensitivity than MP2RAGE, e.g., in Müller et al. (2022) its reported that ${\text{FLAWS}}_{HCO}$ and ${\text{FLAWS}}_{MIN}$ detect in median per patient 4.5 CL compared to 3 detected on MP2RAGE (p-value <0.001).

Artificial Intelligence (AI) methods have shown promise in easing the task of lesion detection. However, these efforts have predominantly focused on WML, with only a few attempts to address CL detection, either along with WML under the general lesion category (La Rosa et al., 2020) or using 7T MRI (La Rosa et al., 2022). This partly explains the modest integration of automated tools into MS clinical pipelines (Spagnolo et al., 2023).

These promising findings steer the aim for our work: assess the capabilities of state-of-the-art DL models when employing the FLAWS sequence for CL detection and segmentation. Our approach focuses on the following objectives: (1) To evaluate how the inclusion of FLAWS sequence enhances CL automatic assessment compared to using MP2RAGE alone, (2) To assess the model performance in correctly identifying false negative and false positives findings in single-rater annotations, and (3) To investigate the capability of DL models trained on FLAWS-derived information to assess CL when only common research sequences (e.g., MP2RAGE) or standard clinical sequences (e.g., MPRAGE) are available, measuring the model’s ability to perform accurately when the test data differs (shift) from the training data.

By addressing these objectives, we aim to rigorously evaluate the performance of DL models in a low data regime as often presented in clinical contexts. Our aim is to support the early integration of these advanced DL models in clinical research settings, enhancing CL assessment by medical doctors, and, consequently, improving MS diagnosis and monitoring.

## 2 Materials and methods

For this study, institutional review board approval and written informed consent were obtained from all participants prior to enrollment.

### 2.1 Study design

This prospective, exploratory study evaluates the feasibility of leveraging FLAWS-derived contrasts for automatic CL detection and segmentation in MS, aiming to enhance diagnosis and disease monitoring.

This study uses the state-of-the-art *nnU-Net* framework v2.0 (Isensee et al., 2021) for semantic segmentation, subsequently instantiated to obtain individual CL. We develop DL models for various contrasts and their derived annotations combinations. Through comparative analysis, we assess the impact of FLAWS-derived contrasts on automated CL assessment in MS patients, evaluating their effectiveness both when these contrasts are directly accessible and when applying FLAWS-trained models to non-FLAWS data.

### 2.2 Dataset description

MRI scans from 204 MS patients were included. The scans were acquired at two centers: University Hospital Basel (hereafter Hospital A; N = 163) between 2020–2022 and Lausanne University Hospital (hereafter Hospital B; N = 41) between 2017–2019. Imaging was performed on *Prisma* (Hospital A) and *Trio* (Hospital B) 3.0 T MRI scanners (Siemens Healthineers, Erlangen, Germany). All patients met the 2017 McDonald criteria (Thompson et al., 2018) for MS diagnosis. Detailed cohort characteristics and imaging protocols are summarized in Table 1.

The dataset was divided into:

• **Internal subset**: MRI acquisitions from a single scanner at Hospital A, representing the in-domain data on which model development was based.
  1. *A-MP2+FL* (N = 69): Includes both FLAWS sequence (with derived contrasts: ${\text{FLAWS}}_{HCO}$ and ${\text{FLAWS}}_{MIN}$) and MP2RAGE. This cohort was partitioned into training /validation (N = 39) and testing (N = 30) subsets with stratification to maintain consistent CL prevalence (see subsection Appendix A.4), age distribution, and sex ratio.
  2. *A-MP2* (N = 94): Independent cohort comprising patients with only MP2RAGE scans, acquired as part of a different study (Granziera, 2018).
• **External subset**: MRI acquisitions from Hospital B, representing out-of-distribution data for generalizability assessment (N = 41).
  3. *B-MP2* and *B-MPR*: This external testing cohort introduces two significant distributional shifts: (1) different MRI scanner hardware, and (2) altered acquisition parameters. *B-MP2* comprises scans acquired using an early implementation of the MP2RAGE sequence with protocol parameters that differ from current standardized implementations (see Table 1). *B-MPR* comprises clinically standard MPRAGE scans, introducing an additional sequence-type shift to evaluate cross-sequence generalization. CL prevalence can be checked in subsection Appendix A.4.

For clarity and consistency throughout this paper, we adopt a standardized nomenclature for dataset subsets as follows.

(1) $${\text{Site}}_{\text{Phase-Annotation}}^{\text{Modality}},$$

where “Site” denotes the hospital of origin (A or B); “Modality” represents the imaging sequences used (MP2 for MP2RAGE, FL for the FLAWS contrasts, MP2+FL for both, or MPR for MPRAGE); “Phase” indicates dataset utilization (Train or Test); and “Annotation” specifies the annotation methodology (see Section 2.4). For example, ${\text{A}}_{\text{Test-Consensus}}^{\text{MP2+FL}}$ refers to the testing subset from Hospital A with both MP2RAGE and FLAWS modalities, using consensus annotations from multiple raters.

### 2.3 Image preprocessing

In the *A-MP2+FL* dataset, MP2RAGE images for each patient were linearly registered to the corresponding ${\text{FLAWS}}_{HCO}$ using *Advanced Normalization Tools* (ANTs) v.2.3.1 (Avants et al., 2009). The whole dataset underwent skull stripping using SynthStrip v2.0 (Hoopes et al., 2022). In addition, within the *nnU-Net* framework z-score normalization was automatically performed.

**Table 1:** Summary of the available datasetand their partitions following the notation defined in Eq. (1). Values expressed as mean ± standard deviation or median [interquartile range]. EDSS: Expanded Disability Status Scale. RRMS: Remitting Relapsing Multiple Sclerosis. SPMS: Secondary Progressive Multiple Sclerosis. PPMS: Primary Progressive Multiple Sclerosis.

### 2.4 Reference standard: Cortical Lesions annotations

All images were manually annotated by expert neurologists, each with 6–22 years of experience in MS neuroimaging assessment, using ITK-SNAP (Yushkevich et al., 2006). The raters were blinded to the clinical status of the patients, and the CL delimitation followed the guidelines established in recent MS imaging literature (Fartaria et al., 2016, Müller et al., 2022).

CL for all subjects in the ${\text{A}}^{MP2+FL}$ dataset (N = 69) were manually segmented by J.M., 6 years of experience, (hereafter *R1*) on MP2RAGE, ${\text{FLAWS}}_{HCO}$, and ${\text{FLAWS}}_{MIN}$. This approach enabled the creation of two types of reference standards for subsequent model development and evaluation (see Fig. 1 upper part): *R1 union*, corresponding to the union of segmentations from all three sequences, and *R1 partial*, derived from a subset of sequences—either solely from MP2RAGE annotations or from the combined ${\text{FLAWS}}_{HCO}$ and ${\text{FLAWS}}_{MIN}$ annotations always obtained independently on each contrast. To assess model capabilities to automatically detect overlook and misidentified CL, the segmentations of 30 out of the 69 patients in the dataset were reviewed by two additional experts (C.T. and A.C., with 6 and 8 years of experience, respectively, referred to as *R2* and *R3*). The review process followed a systematic approach: first, the experts independently verified each previously identified lesion as a genuine CL. Second, they refined the delineation of existing lesions where necessary, and added any lesions that had been overlooked by the initial annotator. To minimize bias, this process was conducted separately for each modality (MP2RAGE, FLAWS-HCO, and FLAWS-MIN) with a two-week interval between each modality’s assessment. Finally, the refined delineations from all three modalities were merged into a single mask per patient and jointly reviewed by all three raters to ensure consistency and eliminate any potential spurious lesions (notably, no lesions were removed during this consensus review). This rigorous multi-rater process resulted in the final reference standard labeled as *Consensus union*, which give us ${\text{A}}_{\text{Test-Consensus}}^{\mathrm{MP2+FL}}$, ${\text{A}}_{\text{Test-Consensus}}^{\mathrm{MP2}}$ and ${\text{A}}_{\text{Test-Consensus}}^{\mathrm{FL}}$.

For the *A-MP2* dataset, manual segmentation was performed by *R3*. based exclusively on the MP2RAGE contrast., given ${\text{A}}_{\text{Test-R3}}^{\text{MP2}}$

For out-of-distributions testing data in Hospital B (i.e., *B-MP2* and *B-MPR*), C.G. (i.e., R4) 22 years of experience conducted the manual segmentation using MP2RAGE, which give us the subsets ${\text{B}}_{\text{Test-R4}}^{\text{MP2}}$ and ${\text{B}}_{\text{Test-R4}}^{\text{MPR}}$.

### 2.5 Model development

We developed, trained and evaluated five segmentation-supervised models using data from 39 subjects (19.12% of total cohort) with both MP2RAGE and FLAWS sequences available employing just *R1* annotations (i.e., ${\text{A}}_{\text{Train-R1union}}^{\text{MP2+FL}}$ and ${\text{A}}_{\text{Train-R1partial}}^{\text{MP2+FL}}$). Our model development strategy explored the effectiveness of different sequence combinations and annotation approaches, reflecting common clinical scenarios where novel sequences are initially tested on limited, partially annotated datasets.

We implemented five models varying their input sequences and potential existing annotations. To distinguish among the models we follow the nomenclature previously defined for subsets by adding “M −” prefix to them to highlight that the model was training employing such specific partition.

1. $M{\text{-A}}_{Train-R1union}^{MP2+FL}$: Both FLAWS contrasts and MP2RAGE, with *R1 union* annotations
2. $M{\text{-A}}_{Train-R1union}^{FL}$: Only FLAWS contrasts, with *R1 union* annotations
3. $M{\text{-A}}_{Train-R1partial}^{FL}$: Only FLAWS contrasts, with *R1 partial* (*FLAWS*) annotations
4. $M{\text{-A}}_{Train-R1union}^{MP2}$: Only MP2RAGE, with *R1 union* annotations
5. $M{\text{-A}}_{Train-R1partial}^{MP2}$: Only MP2RAGE, with *R1 partial (MP2RAGE)* annotations

Table 2 summarizes each model’s configuration and its application in the experiments detailed in Experimental Design, which align with our study objectives.

Each model was built using the *nnU-Net* framework (Isensee et al., 2021), which automatically configures image intensity normalization during preprocessing (see Image Preprocessing), network architecture and training. No additional post-processing was required for any model. For all models, the *3D full-resolution* architecture was employed.

**Table 2:** Summary of model configurations, training data, and their application across experiments. Each experiment corresponds to a specific study objective: Experiment 1 evaluates FLAWS contribution to CL assessment (Objective 1), Experiment 2 assesses the detection of single-rater false positive and negative findings (Objective 2), and Experiment 3 investigates CL assessment using common clinical sequences employing models trained with FLAWS annotations (Objective 3).

The training process involved:

• Extensive data augmentation (details in Isensee et al. (2021) Supplementary Notes 4).
• 1000 training epochs for five-folds, each with different model weight initializations to enhance generalization.
• Post-training ensemble creation by voxel-wise averaging of softmax probabilities from the final layer across the five models.
• Final binary semantic segmentation obtained by thresholding the ensemble’s voxel-wise softmax probabilities, classifying voxels based on the channel (CL or rest) with the highest probability.

To further refine the training process, we modified the *nnU-Net* learning strategy by:

• Employing the ADAM optimizer with an initial learning rate of $3\times 10^{-4}$ (Kingma and Ba, 2014).
• Implementing *Blob Loss* (Kofler et al., 2023), which is more appropriate for the sparse nature of CL, in place of the default loss function in *nnU-Net*.
• Utilizing a deep supervision strategy with *Blob Loss*, combining Dice Similarity Coefficient (DSC) loss (Sudre et al., 2024) and Cross Entropy loss for each detected blob.

All models were trained using an NVIDIA RTX3090 (24Gb vRAM) GPU.

### 2.6 Experimental design

We conducted three main experiments to address our objectives:

1. We evaluated whether the FLAWS sequence enhances automatic CL detection through two distinct mechanisms: (1) as direct model input (either alone or combined with MP2RAGE), and (2) through models trained on annotations derived from FLAWS-enhanced visualization. For mechanism (1), we compared models trained on FLAWS alone ($M\text{-}{\text{A}}_{\text{Train-R1Union}}^{\text{FL}}$) versus MP2RAGE alone ($M\text{-}{\text{A}}_{\text{Train-R1Union}}^{\text{MP2}}$) versus their combination ($M\text{-}{\text{A}}_{\text{Train-R1Union}}^{\text{MP2+FL}}$). For mechanism (2), we compared models trained using either comprehensive annotations from all contrasts (*R1-Union*) or contrast-specific partial annotations (*R1-Partial*). This experiment utilized the internal testing subset, ${\text{A}}_{\text{Test-R1}}^{\text{MP2+FL}}$, comprising 30 held-out subjects from Hospital A not included in model training or validation, allowing for unbiased evaluation of detection performance across different input modality and annotation combinations.

2. Evaluation of the models’ ability to improve CL detection accuracy by comparing their performance against expert manual consensus. This experiment assesses the models’ capacity to identify CL overlooked by a single rater and to correctly exclude false positives initially misidentified as CL. The assessment compares the models’ predictions (trained on *R1 union* annotations, $M\text{-}{\text{A}}_{\text{Train-R1Union}}^{\text{MP2+FL}}$, $M\text{-}{\text{A}}_{\text{Train-R1Union}}^{\text{FL}}$, and $M\text{-}{\text{A}}_{\text{Train-R1Union}}^{\text{MP2}}$) against both single-rater annotations (i,e., ${\text{A}}_{\text{Test-R1Union}}^{\text{MP2+FL}}$, ${\text{A}}_{\text{Test-R1Union}}^{\text{MP2}}$ and ${\text{A}}_{\text{Test-R1Union}}^{\text{FL}}$) and the consensus reference standard (i.e., ${\text{A}}_{\text{Test-Consensus}}^{\text{MP2+FL}}$, ${\text{A}}_{\text{Test-Consensus}}^{\text{MP2}}$ and ${\text{A}}_{\text{Test-Consensus}}^{\text{FL}}$) for the 30 internal testing subjects. This design enables objective measurement of detection performance relative to different reference standards, providing insight into the models’ effectiveness compared to individual expert assessment.

3. To assess the generalizability of models to more widely available clinical sequences, we evaluated whether models trained on MP2RAGE but benefiting from FLAWS-derived annotations could effectively detect CLs in standard clinical sequences. Specifically, we tested models trained exclusively on MP2RAGE inputs but using either comprehensive annotations from all contrasts (i.e., $M\text{-}{\text{A}}_{\text{Train-R1Union}}^{\text{MP2}}$) or only MP2RAGE-derived annotations ($M\text{-}{\text{A}}_{\text{Train-R1Partial}}^{\text{MP2}}$). We applied these models to three independent test datasets: (a) The internal MP2RAGE-only subset (${\text{A}}_{\text{Test-R3}}^{\text{MP2}}$, N = 94 subjects) from Hospital A, (b) external MP2RAGE dataset (${\text{B}}_{\text{Test-R4}}^{\text{MP2}}$, N = 41 subjects) acquired on a different scanner with protocol variations and, (c) its corresponding clinical MPRAGE images (${\text{B}}_{\text{Test-R4}}^{\text{MPR}}$) from the same Hospital B subjects.

The external datasets from Hospital B exhibited significant distributional shifts compared to the training data (see Table 1) (Castro et al., 2020), allowing us to evaluate the models’ robustness to scanner differences, protocol variations, and entirely different sequence types.

### 2.7 Evaluation and statistical analysis

Model evaluation adhered to the *Metrics Reloaded* framework (Maier-Hein et al., 2024), treating the task as instance segmentation to account for the sparse nature of CL. We instantiated reference standard annotations and predictions into CL blobs using the Connected Component algorithm with 18-connectivity (Lehmann, 2007). To match the reference and predict CL, we employed a Mask Intersection over Union (IoU) threshold of 10% and the Greedy by Score assignment strategy.

For all experiments, performance assessment focused on two key metrics: the $F_{1}$-score for detection and the DSC for overlap between CL positive instances. The $F_{1}$ score, which ranges from 0 to 1, provides a balanced measure of the model’s precision (correctness of positive predictions) and recall (ability to detect all positive cases). A higher $F_{1}$-score indicates better overall detection performance. These metrics are summarized using boxplots to illustrate the distribution of performance across different models and conditions.

Statistical analysis for all experiments included paired Wilcoxon signed-rank tests to assess differences between models in averaged per-patient $F_{1}$ and DSC. To control for multiple comparisons, we applied the Benjamini–Hochberg correction.

Additionally, for each experiment, we conducted correlation analyses, created corresponding Bland-Altman plots for agreement, and calculated the intra-class correlation coefficient (ICC). ICC is specifically applied to lesion count per patient comparison to assess reliability of quantitative measurements, as recommended for continuous variables. We deliberately avoided applying ICC to binary detection tasks (i.e., whether specific lesions were identified), where metrics like F1-score are more appropriate according to biomedical image analysis guidelines (Maier-Hein et al., 2023). The ICC model was selected following guidelines by Koo and Li (2016) and McGraw and Wong (1996), using a two-way mixed-effect model with “single-rater” type defined for absolute agreement (i.e., ICC(2,1) as classified in Shrout and Fleiss (1979)). For Experiments 1 and 3, we analyzed the correlation between the number of detected lesions and the reference standard. Bland-Altman plots were used to assess agreement and potential biases in lesion count detection. For Experiment 2, we performed correlation analysis on the $F_{1}$ scores obtained using single-expert annotations versus consensus annotations. The corresponding Bland-Altman plot evaluated the differences in $F_{1}$ scores between these two annotation methods, providing insight into the consistency and potential biases in model performance across different reference standards. The choice between Pearson or Spearman correlation coefficients was based on the normality of data distribution in each case. All statistical analyses and visualizations were conducted using R (version 4.3.3) (Team, 2021) with packages *rstatix* (version 0.7.2) (Kassambara, 2023) and *irr* (version 0.84.1) (Gamer et al., 2019).

## 3 Results

Table 1 summarizes demographic characteristics for the dataset.

Results are shown as mean ± standard deviation or median [interquartile range]. Intraclass correlation coefficient (ICC) values are reported with 95% confidence intervals (complete ICC statistics for all experiments can be found in Appendix A).

### 3.1 Experiment 1: FLAWS contribution to CL detection

**Fig. 2:**
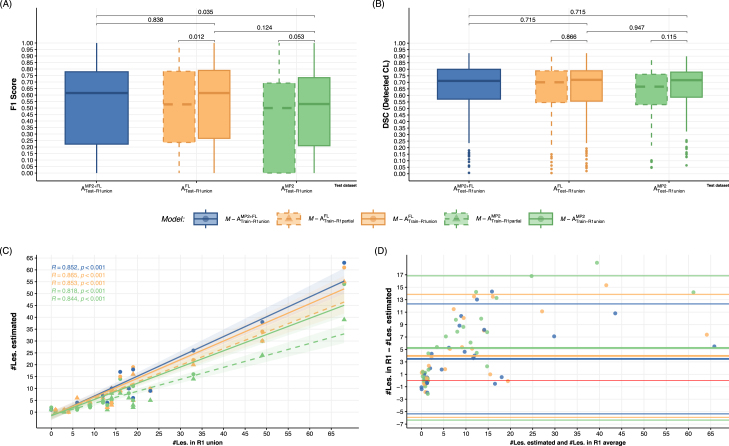
Performance comparison of the five implemented models. Solid lines and circle-points indicate models trained with *R1 Union* annotations; dashed lines and triangle-points correspond with *R1 Partial* . (A) Box plots depicting CL detection performance ($F_{1}$ score). (B) Dice Similarity Coefficient (DSC) for overlap assessment of detected CL. The adjusted p-values corresponding with Wilcoxon signed-rank test are shown. (C) Spearman correlation between estimated lesion count and reference standard when training on *R1 union* and *R1 partial* annotations and testing on union. R coefficients and corresponding p-values are shown for each regression line. (D) Bland-Altman plot showing the agreement between estimated and reference lesion counts for models trained and tested on union annotations. Additional results comparing performance on partial annotation test sets are provided in subsection Appendix A.1.

This experiment evaluated the performance of models trained with either FLAWS, MP2RAGE or jointly FLAWS and MP2RAGE sequences as inputs, using both partial and union annotations and respectively test on ${\text{A}}_{\text{train or test-R1union}}^{\text{MP2+FL or MP2 or FL}}$. Results are presented in Fig. 2.

Analysis of $F_{1}$ scores (Fig. 2A) revealed that models trained with annotations from all contrasts (*R1 union*, solid contour lines) consistently outperformed those trained with partial annotations (dashed contour lines), regardless of input sequences and reference annotations. Among models trained with R1-Union annotations, the combined $M{\text{-A}}_{\text{Train-R1Union}}^{\text{MP2+FL}}$ model achieved the highest median $F_{1}$ score (0.615 [0.339–0.840]), followed by the FLAWS-only model $M{\text{-A}}_{\text{Train-R1Union}}^{\text{FL}}$ (median $F_{1}=0.611\left[0.225-0.790\right]$), both outperforming the MP2RAGE-only model $M{\text{-A}}_{\text{Train-R1Union}}^{\text{MP2}}$ (median $F_{1}=0.531\left[0.029-0.738\right]$).

Statistical analysis revealed a significant difference between the combined model and MP2RAGE-only model (p = 0.035), while the difference between the FLAWS-only model and MP2RAGE-only model showed a trend but did not reach statistical significance (p = 0.124). Notably, the combined model and FLAWS-only model performed similarly (p = 0.838), suggesting that FLAWS sequences already capture most of the relevant information for CL detection. Detailed analyses comparing models trained on partial annotations are provided in the Supplementary Material (Appendix A.1).

DSC analysis (Fig. 2B) indicated good overlap between true detected CL across all models (i.e., $M{\text{-A}}_{R1-union}^{MP2+FL}$ median DSC=0.711±0.20), with no statistically significant differences observed.

The correlation between detected lesions and the reference standard is illustrated in Fig. 2C. The $M{\text{-A}}_{R1-union}^{MP2+FL}$ demonstrated strong agreement (R=0.852, p<0.001, ICC=0.929[0.718,0.974]), closely followed by the $M{\text{-A}}_{R1-union}^{FL}$ model (R=0.86, p<0.001, ICC=0.906[0.640,0.966]). The MP2RAGE-based model, $M{\text{-A}}_{R1-union}^{MP2}$, showed a lower correlation (R=0.82, p<0.001, ICC=0.864[0.496,0.950]) and a tendency to overestimate CL count.

The Bland-Altman plot (Fig. 2(D)) provides further insight into the agreement between the estimated number of lesions and the reference standard. The $M{\text{-A}}_{R1-union}^{MP2+FL}$ model demonstrates the least bias, with a mean difference close to zero (i.e., 3.48±4.51 vs 3.97±5.05 for the $M{\text{-A}}_{R1-union}^{FL}$ and 5.24±5.92 for $M{\text{-A}}_{R1-union}^{MP2}$). All models show a tendency to underestimate lesion count, especially in cases with higher lesion loads. The limits of agreement are narrowest for the $M{\text{-A}}_{R1-union}^{MP2+FL}$ model, indicating better overall agreement with the reference standard across the range of lesion counts.

### 3.2 Experiment 2: Model performance against expert consensus

This experiment compared model performance when trained and tested on single annotator segmentation maps (*R1 union*) versus testing on expert consensus annotations (*consensus*).

**Fig. 3:**
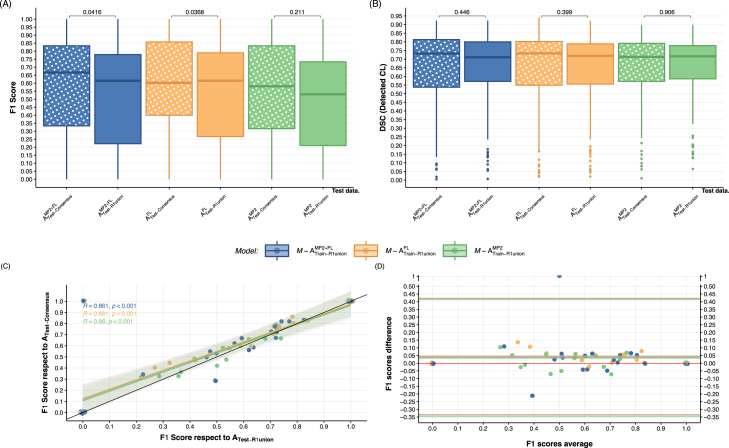
Model performance comparison using single expert (*R1 union*) vs. expert consensus (*consensus*) annotations. (A) Box plots depicting CL detection performance ($F_{1}$ score). (B) DSC for overlap assessment of detected CL. (C) Correlation between $F_{1}$ scores obtained using *R1 union* and *consensus union* annotations. (D) Bland-Altman plot showing the agreement between $F_{1}$ scores from *R1 union* and *consensus union* annotations. Solid box plots represent *R1 union* annotations; dotted box plots represent *consensus union* annotations. R coefficients and corresponding p-values are shown for each correlation.

Analysis of $F_{1}$ scores (Fig. 3A) revealed improved CL detection for all three input contrast combinations models (i.e, $M{\text{-A}}_{Train-R1union}^{MP2+FL}$, $M{\text{-A}}_{Train-R1union}^{MP2}$ and $M{\text{-A}}_{Train-R1union}^{FL}$) when tested against the *consensus* annotations (i.e., ${\text{A}}_{Test-consensus}^{MP2+FL}$, ${\text{A}}_{Test-consensus}^{MP2}$ and ${\text{A}}_{Test-consensus}^{FL}$) compared to the *R1 union* (i.e., ${\text{A}}_{Test-R1union}^{MP2+FL}$, ${\text{A}}_{Test-R1union}^{MP2}$ and ${\text{A}}_{Test-R1union}^{FL}$). The $M{\text{-A}}_{Train-R1union}^{MP2+FL}$ model showed the most significant improvement, with the median $F_{1}$-score increasing from 0.615 ± 0.361 to 0.667 ± 0.366 (p=0.0416). The $M{\text{-A}}_{Train-R1union}^{FL}$ model also demonstrated improvement (p=0.0368), with median $F_{1}$ scores increasing from 0.541±0.36 to 0.585±0.36 for *R1 union* and *consensus union* annotations, respectively. The $M{\text{-A}}_{Train-R1union}^{MP2}$ model showed a smaller, non-significant improvement from mean $F_{1}=0.503\pm 0.36$ to $F_{1}=0.537\pm 0.36$ (p=0.211).

DSC analysis (Fig. 3B) showed no significant differences in overlapping performance for detected lesions between *R1 union* and *consensus union* annotations, suggesting that while the models detect more lesions when compared to the consensus, the accuracy of segmentation remains consistent.

The correlation analysis between $F_{1}$ scores obtained using *R1 union* and *consensus union* annotations (Fig. 3C) demonstrated strong agreement across all models. The $M{\text{-A}}_{Train-R1union}^{MP2+FL}$ and $M{\text{-A}}_{Train-R1union}^{FL}$ models both showed identical correlation coefficients (R=0.86, p<0.01, ICC=0.859[0.724,0.931]), while the $M{\text{-A}}_{Train-R1union}^{MP2}$ model showed a slightly lower but still strong correlation (R=0.86, p<0.001, ICC=0.858[0.722,0.930]). This high correlation indicates consistent relative performance across annotation methods, regardless of the absolute $F_{1}$ scores.

The Bland-Altman plot (Fig. 3D) provides insight into the systematic differences between $F_{1}$ scores obtained using *R1 union* and *consensus union* annotations. All models show a positive mean difference, indicating consistently higher $F_{1}$ scores when evaluated against the *consensus union*. The $M{\text{-A}}_{Train-R1union}^{FL}$ model demonstrates the largest mean difference (0.045), followed by the $M{\text{-A}}_{Train-R1union}^{MP2+FL}$ model (0.041), and the $M{\text{-A}}_{Train-R1union}^{MP2}$ model (0.034). The limits of agreement are equally narrow for $M{\text{-A}}_{Train-R1union}^{MP2+FL}$ and $M{\text{-A}}_{Train-R1union}^{FL}$.

### 3.3 Experiment 3: Generalization to clinical sequences

**Fig. 4:**
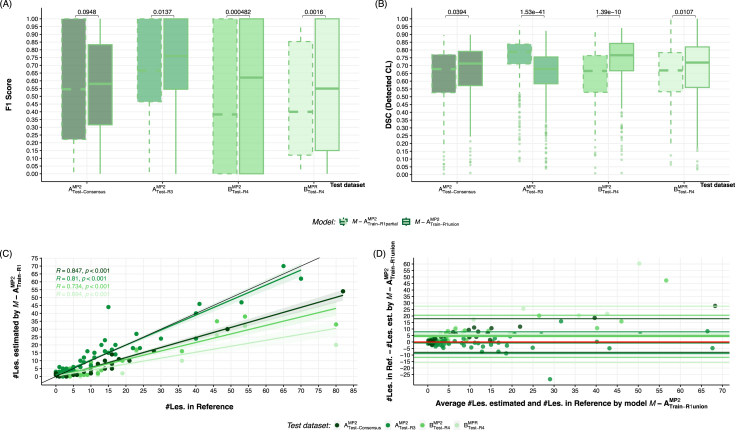
Performance comparison of models trained on MP2RAGE sequence as inputs employing annotations derived from MP2RAGE alone ($M{\text{-A}}_{\text{Train-R1Partial}}^{\text{MP2}}$) or all contrasts ($M{\text{-A}}_{\text{Train-R1Union}}^{\text{MP2}}$), evaluated on three independent test datasets: (a) the internal test subset (${\text{A}}_{\text{Test-Consensus}}^{\text{MP2}}$), (b) internal MP2RAGE-only dataset (${\text{A}}_{\text{Test-R3}}^{\text{MP2}}$), (c) external MP2RAGE dataset (${\text{B}}_{\text{Test-R4}}^{\text{MP2}}$), and (d) corresponding clinical MPRAGE images (${\text{B}}_{\text{Test-R4}}^{\text{MPR}}$). (A) Box plots depicting CL detection performance. (B) DSC for overlap assessment of detected CL. Adjusted p-values from Wilcoxon signed-rank tests are shown. (C) Spearman correlation between estimated lesion count and reference standard for different datasets. (D) Bland-Altman plot showing the agreement between estimated and reference lesion counts for the $M{\text{-A}}_{\text{Train-R1Union}}^{\text{MP2}}$ model across all test datasets.

This experiment evaluated the performance of two models, $M{\text{-A}}_{Train-R1union}^{MP2}$ and $M{\text{-A}}_{Train-R1partial}^{MP2}$ when applied to different independent test datasets comprising MP2RAGE and MPRAGE sequences. Results are presented in Fig. 4.

Analysis of $F_{1}$ scores (Fig. 4A) revealed that the *R1 union* model ($M{\text{-A}}_{Train-R1union}^{MP2}$), trained using annotations derived from all available sequences, consistently outperformed the partial model ($M{\text{-A}}_{Train-R1partial}^{MP2}$) in lesion detection across all test datasets. This performance advantage was particularly pronounced in the external out-of-domain dataset, ${\text{B}}_{Test-R4}^{MP2}$, where $M{\text{-A}}_{Train-R1union}^{MP2}$ achieved a median $F_{1}$ score of 0.621±0.406 compared to 0.383±0.419 for $M{\text{-A}}_{Train-R1partial}^{MP2}$ (p<0.001).

Importantly, for the clinical MPRAGE sequence (${\text{B}}_{Test-R4}^{MPR}$) external testing dataset , the model based in *R1 union* annotation, obtained a median $F_{1}$ score of 0.55±0.356, while its partial counterpart achieved only 0.40±0.44). Notably, the $M{\text{-A}}_{Train-R1union}^{MP2}$ model’s performance on ${\text{B}}_{Test-R4}^{MP2}$ was comparable to its performance on the held-out subjects from the internal ${\text{A}}_{Test-consensus}^{MP2}$ dataset ($F_{1}=0.581\pm 0.36$), suggesting robust generalization to external data.

This *R1 union* model demonstrated superior detection capabilities on the internal test dataset ${\text{A}}_{Test-R3}^{MP2}$, achieving a median $F_{1}$ score of 0.754±0.34.

DSC analysis (Fig. 4B) revealed a more nuanced picture of segmentation accuracy. Interestingly, the $M{\text{-A}}_{Train-R1partial}^{MP2}$ model showed better DSC results for the internal *MP2RAGE* dataset, a trend not observed in the other test datasets.

Given the consistently superior detection performance of the $M{\text{-A}}_{\text{Train-R1Union}}^{\text{MP2}}$ model, further correlation and agreement analyses focused exclusively on this model. The correlation analysis (Fig. 4C) provides insight into the relationship between estimated and reference lesion counts across datasets. The *union* model showed strong correlations for both the *in-domain* ${\text{A}}_{Test-R3}^{MP2}$ dataset (R=0.81, p<0.001, ICC=0.950[0.926,0.967]) and the ${\text{A}}_{Test-consensus}^{MP2}$ (R=0.85, p<0.001, ICC=0.851[0.526,0.942]). The correlation was weaker for the external ${\text{B}}_{Test-R4}^{MP2}$ dataset (R=0.73, p<0.001, ICC=0.774[0.544,0.885]) and ${\text{B}}_{Test-R4}^{MPR}$ (R=0.66, p<0.001, ICC=0.581[0.281,0.768]),

The Bland-Altman plot (Fig. 4D), focusing solely on the $M{\text{-A}}_{Train-R1union}^{MP2}$ model, demonstrates the agreement between estimated and reference lesion counts.

### 3.4 Qualitative results

**Fig. 5:**
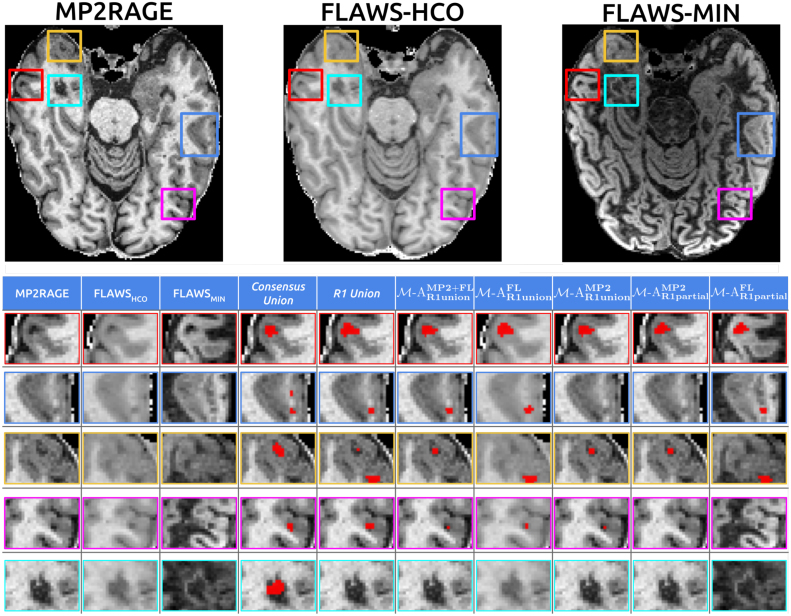
Examples of different CL across available contrasts (top and zoomed views in the first three grid columns), manual segmentations (*Consensus* and *R1 Union* columns), and predictions from each model (Model Development) in the last five grid columns.

Fig. 5 presents manual (fourth and fifth columns) and automatic segmentation predictions for each model (last five columns in the grid) across various lesion types. The figure illustrates several key observations: In the first row (red square), all models and manual segmentations concur on lesion identification. The second row (dark blue rectangle) showcases a case where the *Consensus* annotations reveal a previously unidentified lesion. Models relying solely on MP2RAGE input fail to detect both lesions highlighted in the revision, while FLAWS-based models identify one lesion, consistent with the *R1 Union* annotation, regardless of the training annotation type. Conversely, the third row (yellow square) demonstrates a scenario where MP2RAGE-based models align with the consensus manual annotation, detecting one lesion, while FLAWS-based models identify two lesions as per the *R1 Union* reference. The fourth row (violet square) highlights the performance of models utilizing all available annotation information. Only models trained on *R1 union* annotations correctly identify the small lesion presented, in concordance with Experiment 2. The final row illustrates a case where a lesion, initially undetected in the single manual annotation, was subsequently missed by all models.

Notably, the $M{\text{-A}}_{\text{Train-R1Union}}^{\text{MP2+FL}}$ model consistently shows the closest alignment with the consensus annotations across various scenarios, corroborating the quantitative findings from previous experiments.

## 4 Discussion

This study demonstrates the potential of leveraging FLAWS-derived contrasts for improved automatic CL detection and segmentation in MS patients using DL models. Our findings have several important implications for the field of MS MRI imaging and AI-assisted diagnosis.

### Enhanced CL DL detection with FLAWS.

The integration of FLAWS-derived contrasts significantly improved automatic CL assessment, as demonstrated in Experiment 1 and Experiment 2. Models incorporating FLAWS ($M{\text{-A}}_{\text{Train-R1Union}}^{\text{MP2+FL}}$ and $M{\text{-A}}_{\text{Train-R1Union}}^{\text{FL}}$) consistently outperformed the $M{\text{-A}}_{\text{Train-R1Union}}^{\text{MP2}}$ model, with higher $F_{1}$ scores and stronger correlations to reference standards. Furthermore, models trained on comprehensive annotations from all available contrasts consistently outperformed those trained on partial, sequence-specific annotations, highlighting the value of a multi-contrast approach to CL identification.

An interesting pattern emerged in the statistical comparisons: while the FLAWS-only model showed better median performance than the MP2RAGE-only model, this difference did not reach statistical significance (p=0.124). However, when combining both modalities, the resulting model significantly outperformed the MP2RAGE-only approach (p=0.035). This suggests a complementary information effect, where the addition of MP2RAGE data to FLAWS provides just enough additional relevant features to achieve statistically significant improvement over MP2RAGE alone, despite the combined model performing very similarly to the FLAWS-only model (p=0.838).

Given our relatively limited sample size, these statistical trends should be interpreted cautiously. However, they suggest that FLAWS sequences provide substantial value for CL detection, with the combined approach offering the most robust performance. This finding has important implications for sequence selection in research and potential clinical translation, suggesting that while FLAWS offers significant advantages over MP2RAGE alone, the optimal approach when feasible may be to utilize both sequences and incorporate annotations derived from all available contrasts.

### Enhanced sensitivity of models in CL detection.

Experiment 2 addressed our second objective by evaluating the models’ ability to detect CL potentially overlooked by individual raters. This is particularly relevant given the well-known challenges in CL identification, which depend on both sequence sensitivity and rater expertise (Madsen et al., 2021).

We compared model performance using single-rater annotations against consensus-reviewed annotations. Notably, models, even when trained on single-rater annotations ($M{\text{-A}}_{\text{Train-R1Union}}^{\text{MP2+FL}}$ and $M{\text{-A}}_{\text{Train-R1Union}}^{\text{FL}}$), demonstrated statistically significant better $F_{1}$ scores when evaluated on consensus annotations than on the single-rater annotations in the test set. This suggests that our models can potentially identify CL missed by individual expert raters while maintaining high segmentation accuracy (as indicated by consistent DSC values), showing the model’s ability to extract relevant information beyond the standard exactness available at training. These findings are particularly promising considering the limited dataset size, a common challenge when introducing new imaging sequences. The models’ ability to generalize and potentially outperform single-rater annotations highlights the robustness of the DL approach in CL detection.

### Generalization and transferability to clinical sequences.

Experiment 3 addressed our third objective by evaluating the generalization capabilities of models trained on FLAWS-derived annotations when applied to MP2RAGE and MPRAGE, including both internal and external testing datasets. The $MP2RAGE_{union}$ model, trained on annotations derived from all available contrasts, demonstrated superior performance across different datasets.

Notably, the $M{\text{-A}}_{\text{Train-R1Union}}^{\text{MP2}}$ model achieved higher $F_{1}$ scores compared to its partial counterpart on both internal and external MP2RAGE sequence based datasets. This performance advantage was particularly pronounced on the external, shifted datasets: the ${\text{B}}_{\text{Test-R4}}^{\text{MP2}}$ dataset (median $F_{1}=0.621\pm 0.406$ vs. 0.383±0.419, $p=4.82\times 10^{-4}$, $p=4.82\times 10^{-4}$), and the ${\text{B}}_{\text{Test-R4}}^{\text{MPR}}$ dataset (median $F_{1}=0.55\pm 0.356$ vs. 0.40±0.44, p=0.02). These results suggest a robust model capability for generalization, even under significantly shifted data, as in the case of the clinical MPRAGE sequence.

Interestingly, the model’s performance on the internal ${\text{A}}_{\text{Test-R3}}^{\text{MP2}}$ dataset (94 previously unseen subjects) surpassed its performance on the held-out subjects for internal testing from the *MP2RAGE and FLAWS* dataset. While this finding may seem counterintuitive initially, it can be attributed to the fact that the reference annotations for these datasets were derived solely from the MP2RAGE contrast. This observation reinforces the model’s ability to effectively leverage information learned from FLAWS-derived annotations, even when applied to single-contrast data.

Furthermore, the model’s ability to maintain or even improve performance on MP2RAGE-only data underscores the value of incorporating FLAWS-derived contrasts in the training process. Our results indicate that advanced imaging techniques like FLAWS can enhance CL detection in settings where such sequences are not available, thereby potentially bridging the gap between research capabilities and clinical practice in CL detection through label transfer knowledge.

### Limitations and future directions.

While our study yields promising results, it is crucial to acknowledge its limitations. The training set, though realistically sized for a novel imaging biomarker study, is relatively small. This, coupled with the lack of publicly available CL datasets—a consequence of CL recent emergence as an MS imaging biomarker—presents challenges for comprehensive model development and validation.

Our qualitative results also suggest potential inconsistencies in CL annotation criteria between individual raters and consensus reviews (as seen in the last row of Qualitative results). This variability in lesion definition, akin to what machine learning literature terms *concept shift*, highlights the need for standardized annotation protocols, especially for multi-center studies (Nichyporuk et al., 2022). To address this challenge, we have recently conducted additional studies aimed at explaining the features that drive differences in lesion assessment by deep learning models (Molchanova et al., 2025b, Molchanova et al., 2025a). Additionally, our annotation approach differs from traditional inter-rater reliability assessment. Rather than having multiple independent raters score the same dataset, we employed a sequential improvement process where R2 and R3 reviewed and enhanced R1’s initial annotations. This approach was specifically designed to maximize lesion detection sensitivity, as even a single missed CL can significantly impact MS diagnosis and monitoring according to current diagnostic criteria (Thompson et al., 2018). For the external validation dataset, annotations were performed by R4 independently, without direct inter-rater agreement assessment with other raters. This reflects a broader challenge in the field, where CL assessment using conventional sequences has historically shown variable agreement (Cohen κ≈0.50−0.69 for some advanced techniques (Madsen et al., 2021) and κ=−0.36 for conventional sequences like MPRAGE (Nielsen et al., 2012)).

To address these limitations and further advance the field, we propose several directions for future research. Validation on larger multi-center datasets will be crucial to rigorously assess model generalizability across diverse patient populations and imaging protocols. The development of standardized, publicly available CL datasets would facilitate benchmarking and collaborative research in this emerging field. To facilitate this transition and promote further research, we have made our trained models available through DockerHub (https://hub.docker.com/r/petermcgor/corles-seg-flaws-based), aiming to accelerate the development and validation of AI-assisted CL detection tools across different clinical and research environments.

### Clinical impact.

This work addresses a critical need in MS diagnosis and monitoring, as CL are increasingly recognized as important indicators of disease progression (Beck et al., 2024, Cagol et al., 2024). The identification of even a single CL can be decisive for accurate MS diagnosis according to current criteria (Thompson et al., 2018), making enhanced detection sensitivity particularly valuable in clinical practice. The models’ demonstrated ability to align with consensus annotations while maintaining performance across different sequences suggests potential for practical clinical implementation. Furthermore, our findings substantially contribute to the growing body of evidence supporting the adoption of AI-assisted tools in radiology, particularly for complex tasks such as CL assessment where expert consensus may be impractical or time-consuming.

### Conclusion.

This study demonstrates the significant potential of integrating advanced MRI sequences, specifically FLAWS, with state-of-the-art DL models to enhance CL detection and segmentation in MS. Our work not only advances the technical capabilities of CL detection but also paves the way for more accurate, consistent, and efficient MS assessment through the synergistic combination of novel imaging sequences and AI-assisted analysis.

## CRediT authorship contribution statement

**Pedro M. Gordaliza:** Writing – review & editing, Writing – original draft, Visualization, Validation, Supervision, Software, Methodology, Investigation, Formal analysis, Data curation, Conceptualization. **Jannis Müller:** Writing – review & editing, Writing – original draft, Investigation, Formal analysis, Data curation, Conceptualization. **Alessandro Cagol:** Writing – review & editing, Writing – original draft, Validation, Supervision, Investigation, Funding acquisition, Formal analysis, Data curation. **Nataliia Molchanova:** Writing – review & editing, Writing – original draft, Visualization, Validation, Supervision. **Francesco La Rosa:** Writing – review & editing, Writing – original draft, Visualization, Validation, Supervision. **Charidimos Tsagkas:** Writing – review & editing, Writing – original draft, Visualization, Validation, Supervision, Data curation. **Cristina Granziera:** Writing – review & editing, Writing – original draft, Visualization, Validation, Supervision, Data curation, Conceptualization. **Meritxell Bach Cuadra:** Writing – review & editing, Writing – original draft, Visualization, Validation, Supervision, Formal analysis, Conceptualization.

## Data Availability

The authors do not have permission to share data.

## Appendix A Supplementary data

MMC S1
