# Supplementary material for: Fluid and White Matter Suppression contrasts MRI improves Deep Learning detection of Multiple Sclerosis Cortical Lesions
Source: Neuroimage Clin. 2025 Jul 14;48:103818. doi: 10.1016/j.nicl.2025.103818 (PMC12301823; doi:10.1016/j.nicl.2025.103818)
Supplement: MMC S1 [file mmc1.pdf]

Appendix A. Supplementary Materials

Appendix A.1. Additional Results for Experiment 1

This section presents additional analyses comparing the performance of models trained with partial annotations (R1-Partial) versus those trained with comprehensive annotations across all contrasts (R1-Union).

Figure A.6 compares the performance of models trained with partial annotations ($\mathcal{M}$-$\text{A}^{\text{FL}}_{\text{Train-R1Partial}}$ and $\mathcal{M}$-$\text{A}^{\text{MP2}}_{\text{Train-R1Partial}}$) to their counterparts trained with union annotations when evaluated on their respective partial annotation test sets ($\text{A}^{\text{FL}}_{\text{Test-R1Partial}}$ and $\text{A}^{\text{MP2}}_{\text{Test-R1Partial}}$).

Our results indicate that models trained on union annotations derived from all available contrasts consistently outperformed those trained on partial annotations. This finding suggests that the comprehensive annotation strategy enhances model performance without introducing contrast-specific biases.

Table A.3: Intraclass Correlation Coefficient (ICC) Analysis of Lesion Count Agreement Between Models and Reference Standard (subsection 2.4) for lesion count

Model	ICC	95% CI	p-value	Agreement
$\mathcal{M}$-$\text{A}^{\text{MP2+FL}}_{\text{Train-R1Union}}$	0.929	[0.718, 0.974]	<0.001	Excellent
$\mathcal{M}$-$\text{A}^{\text{FL}}_{\text{Train-R1Union}}$	0.906	[0.640, 0.966]	<0.001	Excellent
$\mathcal{M}$-$\text{A}^{\text{FL}}_{\text{Train-R1Partial}}$	0.864	[0.496, 0.950]	<0.001	Good
$\mathcal{M}$-$\text{A}^{\text{MP2}}_{\text{Train-R1Union}}$	0.847	[0.406, 0.945]	0.001	Good
$\mathcal{M}$-$\text{A}^{\text{MP2}}_{\text{Train-R1Partial}}$	0.681	[0.162, 0.870]	0.007	Moderate

Note: ICC calculated using two-way mixed-effect model with single-rater absolute agreement (ICC(2,1)). Agreement categories based on Koo & Li (2016): < 0.5 = Poor, 0.5-0.75 = Moderate, 0.75-0.9 = Good, > 0.9 = Excellent.

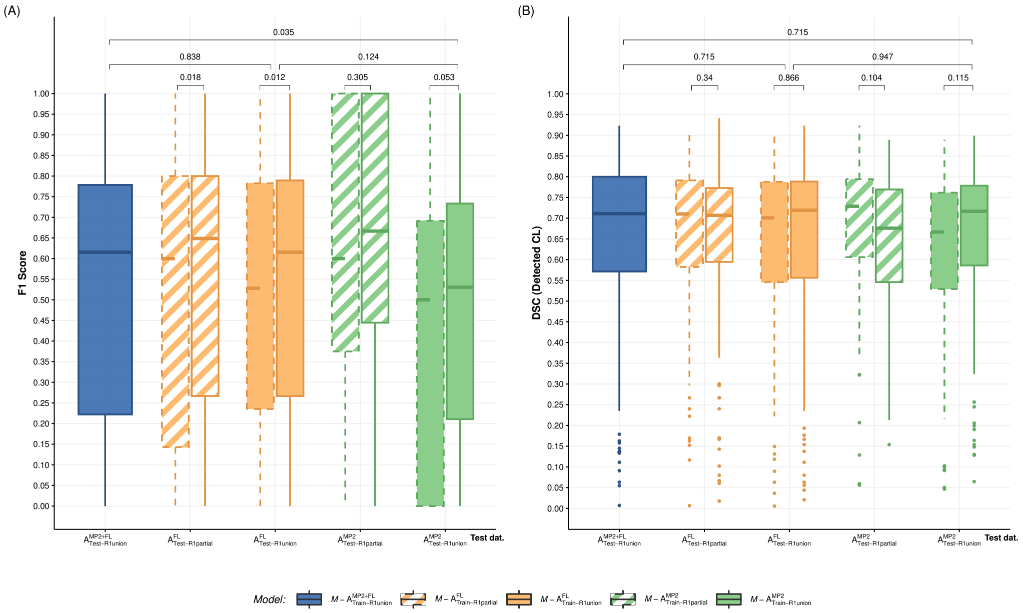

Figure A.6: Additional performance comparison of models trained with different annotation protocols. Models trained with union annotations consistently outperformed those trained with partial annotations when evaluated on their respective test sets. (A) Box plots depicting CL detection performance (F_1 score) on partial annotation test sets. (B) Dice Similarity Coefficient (DSC) for overlap assessment of detected CL. (C) Correlation between estimated lesion count and reference standard. (D) Bland-Altman plot showing the agreement between estimated and reference lesion counts.

Appendix A.2. Additional Results for Experiment 2

Table A.4: Intraclass Correlation Coefficient (ICC) Between Single-Rater and Consensus Annotations for Different Models

Model	ICC	95% CI	p-value	Agreement
$\mathcal{M}$-$A^{\text{MP2+FL}}_{\text{Train-R1Union}}$	0.859	[0.724, 0.931]	<0.001	Good
$\mathcal{M}$-$A^{\text{MP2}}_{\text{Train-R1Union}}$	0.859	[0.724, 0.931]	<0.001	Good
$\mathcal{M}$-$A^{\text{FL}}_{\text{Train-R1Union}}$	0.858	[0.722, 0.930]	<0.001	Good

Note: ICC calculated comparing model performance across single-rater versus consensus annotations, using two-way mixed-effect model with single-rater absolute agreement (ICC(2,1)). Agreement categories: < 0.5 = Poor, 0.5-0.75 = Moderate, 0.75-0.9 = Good, > 0.9 = Excellent.

Appendix A.3. Additional Results for Experiment 3

Table A.5: Intraclass Correlation Coefficient (ICC) Analysis for External Dataset Generalization (subsection 3.3) for Lesion Count employing $\mathcal{M}$-$A^{\text{MP2}}_{\text{Train-R1Union}}$ at inference

Data	ICC	95% CI	p-value	Agreement
$A^{\text{MP2}}_{\text{Test-R3}}$	0.950	[0.926, 0.967]	<0.001	Excellent
$A^{\text{MP2}}_{\text{Test-Consensus}}$	0.851	[0.526, 0.942]	<0.001	Good
$B^{\text{MP2}}_{\text{Test-R4}}$	0.774	[0.544, 0.885]	<0.001	Good
$B^{\text{MPR}}_{\text{Test-R4}}$	0.581	[0.281, 0.768]	<0.001	Moderate

Note: ICC calculated between predicted and reference lesion counts across different test datasets, using two-way mixed-effect model with single-rater absolute agreement (ICC(2,1)). Agreement categories: < 0.5 = Poor, 0.5-0.75 = Moderate, 0.75-0.9 = Good, > 0.9 = Excellent.

Appendix A.4. Lesion Distribution Analysis

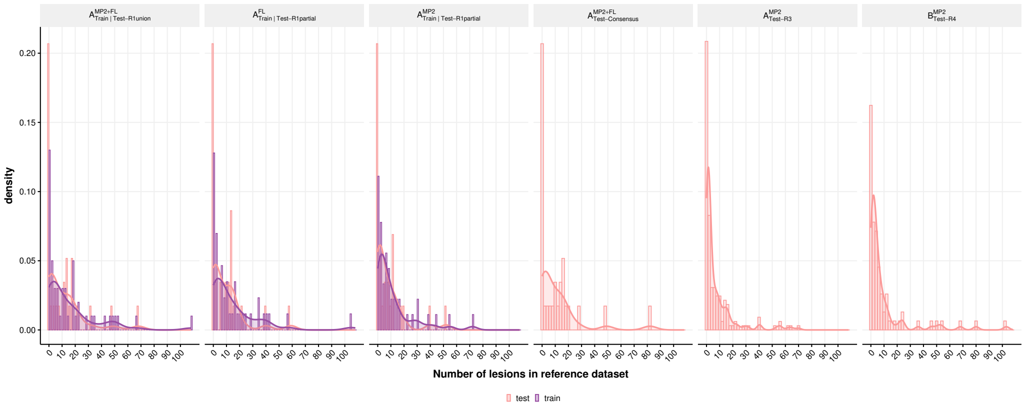

Figure A.7: Distribution of CL count across different datasets and partitions. Histograms show the number of lesion per patients (y-axis) with specific counts (x-axis) for each dataset used in our experiments. The first three panels show the training (purple) and testing (pink) distributions for the $A^{MP2+FL}_{R1union}$, $A^{FL}_{R1partial}$, and $A^{MP2}_{R1partial}$ datasets, demonstrating balanced distributions between training and testing partitions. The remaining three panels show the testing-only datasets: $A^{MP2+FL}_{Consensus}$ (expert consensus annotations), $A^{MP2}_{Test-R3}$ (independent Hospital A dataset), and $B^{MP2}_{Test-R4}$ (external Hospital B dataset). Note the varying distributions across different annotation protocols and sites, highlighting the challenge of generalization across datasets.
